# Prognostic implications of HER2 heterogeneity in gastric cancer

**DOI:** 10.18632/oncotarget.24265

**Published:** 2018-01-18

**Authors:** Shigenobu Motoshima, Koji Yonemoto, Hideki Kamei, Michi Morita, Rin Yamaguchi

**Affiliations:** ^1^ Biostatistics Center, Graduate School of Medicine, Kurume University, Kurume, Fukuoka, Japan; ^2^ Department of Clinical Laboratory, Kokura Medical Center, Kitakyushu, Fukuoka, Japan; ^3^ Advanced Medical Research Center, Faculty of Medicine, University of the Ryukyus, Nishihara, Okinawa, Japan; ^4^ Department of Surgery, Japan Community Health Care Organization Kurume General Hospital, Kurume, Fukuoka, Japan; ^5^ Department of Surgery, Nagasaki University Graduate School of Biomedical Sciences, Nagasaki, Nagasaki, Japan; ^6^ Department of Pathology and Laboratory Medicine, Kurume University Medical Center, Kurume, Fukuoka, Japan

**Keywords:** gastric cancer, HER2, intratumoral heterogeneity, prognosis, trastuzumab

## Abstract

The prognostic implications of human epidermal growth receptor 2 (HER2) heterogeneity in gastric cancer (GC) are not well established. Therefore, the aim of the present study was to determine to the effect of HER2 status on the prognosis of GC patients. We retrieved data on 248 pathologically-confirmed, consecutive patients with primary adenocarcinoma of the stomach or gastro-esophageal junction who underwent surgical resection at Kurume University Medical Center between July 2000 and December 2012. HER2 status was classified as HER2 positive or negative and HER2 heterogeneity or homogeneity. The endpoint was overall survival (OS), which was compared using the generalized Wilcoxon test. HER2 status was positive in 36 patients (14.5%) and negative in 212 patients (85.5%). Among the 36 HER2 positive patients, 25 patients (69.4%) had HER2 heterogeneity and the remaining 11 patients (30.6%) had HER2 homogeneity. Among the 141 patients with stage III or IV disease, the prognosis of the HER2 homogeneity group was significantly worse than that of the HER2 heterogeneity group (*p* = 0.019; median OS 193 and 831 days, respectively). The prognosis was not significantly different between the HER2 positive group and the HER2 negative group (*p* = 0.84; median OS 552 and 556 days, respectively). The present study was conducted with small samples, however, the results of the study suggest that HER2 homogeneity but not HER2 positivity may represent a prognostic indicator in GC.

## INTRODUCTION

The role of human epidermal growth receptor 2 (HER2) in breast cancer (BC) has been widely studied since the late 1980s [1–4] and has recently been established as a biomarker of poor prognosis in BC patients [5, 6], whereas in gastric cancer (GC), the role of HER2 as a biomarker of poor prognosis remains unclear [7]. Moreover, there is a growing concern that HER2 heterogeneity in BC may influence prognosis [8, 9]. However, the effect of HER2 heterogeneity on prognosis of GC patients also remains unclear.

The HER2 targeted agent trastuzumab has been shown to be effective and safe in patients with HER2 positive metastatic BC [10–12] and is now established as a standard initial treatment in HER2 positive BC patients [13–15]. Furthermore, the emerging HER2 targeted agents lapatinib [16] and trastuzumab emtansine (T-DM1) [17] have also been shown to be effective and safe in this patient population. Owing to the effects of these anti-HER2 targeted agents, HER2 positive BC is no longer associated with a poor prognosis [18, 19]. In GC, trastuzumab has also been shown to be effective and safe in the treatment of patients with HER2 positive metastatic or unresectable disease, regardless of conflicting HER2 prognostic values [20]. However, lapatinib and T-DM1 have failed to demonstrate efficacy in HER2 positive GC patients [21–23]. According to Matsuoka *et al*. [24], the efficacy of HER2 targeted agents has been shown to be more limited than expected in GC patients.

It is clear that the clinical implications of HER2 are markedly different between BC and GC patients. With respect to the biological nature of HER2, the frequency of HER2 heterogeneity differs between HER2 positive GC and BC patients, being 45%–79% [25–28] and 11%–40% [8, 9, 29–33], respectively. This difference in frequency may explain the different clinical implications of HER2. In GC, most studies on HER2 heterogeneity have focused on pathological issues [27, 28, 34–40], although two studies to our knowledge have focused on the prognostic implications [25, 26]. These two studies have reported conflicting results concerning the prognosis of HER2 heterogeneity compared with that of HER2 homogeneity. The effect of HER2 heterogeneity on prognosis of GC patients thus remains to be sufficiently elucidated.

The aim of the present study was to determine the differences in the prognosis of GC patients according to HER2 status and thus to clarify the potential of HER2 as a biomarker of prognosis in GC patients with HER2 heterogeneity.

## RESULTS

### Clinicopathological characteristics and HER2 status

Data corresponding to a total of 248 patients were retrieved. HER2 status was positive in 36 patients (14.5%) and negative in 212 patients (85.5%). Among the 36 HER2 positive patients, 25 patients (69.4%) had HER2 heterogeneity and the remaining 11 patients (30.6%) had HER2 homogeneity. The clinicopathological characteristics and HER2 status of the 248 patients are summarized in Table 1. Regarding the quality control of surgically resected samples, HER2 positivity rates did not show any significant difference between the two terms of the study period (July 2000 to December 2006, and January 2007 to December 2012) (*p* = 0.12).

**Table 1:** Clinicopathological characteristics and HER2 status of the enrolled patients

Variables	HER2 positive		Heterogeneity vs. Homogeneity *p*-value^a^	HER2 status		Positive vs. Negative *p*-value^a^	Total (*n* = 248)
	Heterogeneity (*n* = 25)	Homogeneity (*n* = 11)		Positive (*n* = 36)	Negative (*n* = 212)		
Age (years)			0.16^b^			0.052^b^	
Median	70	65		67.5	66		66
Range	47–86	51–81		47-86	38-88		38-88
Advanced age, % (*n*)			0.026^c^			0.072^c^	
≥65 years	88.0 (22)	54.5 (6)		77.8 (28)	62.3 (132)		64.5 (160)
Sex, % (*n*)			0.064^c^			0.35^c^	
Male	60.0 (15)	90.9 (10)		69.4 (25)	61.3 (130)		62.5 (155)
Operative method, % (*n*)			0.46^d^			0.23^d^	
Distal gastrectomy	72.0 (18)	63.6 (7)		69.4 (25)	61.8 (131)		62.9 (156)
Total gastrectomy	28.0 (7)	27.3 (3)		27.8 (10)	32.5 (69)		31.9 (79)
Proximal gastrectomy	0	9.1 (1)		2.8 (1)	4.2 (9)		4.0 (10)
Pylorus-preserving gastrectomy	0	0		0	1.4 (3)		1.2 (3)
Pathologic TNM stage, % (*n*)			0.14^d^			0.10^c^	
I	20.0 (5)	0		13.9 (5)	28.3 (60)		26.2 (65)
II	16.0 (4)	18.2 (2)		16.7 (6)	17.0 (36)		16.9 (42)
III	44.0 (11)	27.3 (3)		38.9 (14)	21.7 (46)		24.2 (60)
IV	20.0 (5)	54.5 (6)		30.5 (11)	33.0 (70)		32.7 (81)
Lauren classification, % (*n*)			0.83^c^			0.0002^c^	
Intestinal type	76.0 (19)	72.7 (8)		75.0 (27)	41.5 (88)		46.4 (115)
Diffuse type	24.0 (6)	27.3 (3)		25.0 (9)	58.5 (124)		53.6 (133)
Depth of tumor invasion, % (*n*)			0.87^d^			0.38^d^	
Mucosa	8.0 (2)	0		5.6 (2)	15.1 (32)		13.7 (34)
Submucosa	8.0 (2)	0		5.6 (2)	3.3 (7)		3.6 (9)
Muscularis propria	12.0 (3)	18.2 (2)		13.9 (5)	11.3 (24)		11.7 (29)
Subserosa	16.0 (4)	27.3 (3)		19.4 (7)	10.9 (23)		12.1 (30)
Serosa and peritoneal cavity	52.0 (13)	54.5 (6)		52.7 (19)	55.2 (117)		54.9 (136)
Adjacent structures	4.0 (1)	0		2.8 (1)	4.2 (9)		4.0 (10)
Lymphatic invasion, % (*n*)			0.53^d^			0.21^c^	
ly0	12.0 (3)	9.1 (1)		11.1 (4)	22.6 (48)		21.0 (52)
ly1	28.0 (7)	9.1 (1)		22.2 (8)	27.8 (59)		27.0 (67)
ly2	32.0 (8)	54.5 (6)		38.9 (14)	25.5 (54)		27.4 (68)
ly3	28.0 (7)	27.3 (3)		27.8 (10)	24.1 (51)		24.6 (61)
Venous invasion, % (*n*)			0.57^d^			0.029^c^	
v0	16.0 (4)	0		11.1 (4)	25.5 (54)		23.4 (58)
v1	28.0 (7)	27.3 (3)		27.8 (10)	28.8 (61)		28.6 (71)
v2	20.0 (5)	36.4 (4)		25.0 (9)	29.5 (62)		28.6 (71)
v3	36.0 (9)	36.4 (4)		36.1 (13)	16.5 (35)		19.4 (48)
IHC score, % (*n*)							
0 or 1+	0	0		0	99.1 (210)		84.7 (210)
2+	36.0 (9)	0		25.0 (9)	0.9 (2)		4.4 (11)
3+	64.0 (16)	100 (11)		75.0 (27)	0		10.9 (27)
DISH, % (*n*) (among the IHC score of 2+ cases)							
Negative	0	0		0	100 (2)		2 (18.2)
Positive	100 (9)	0		100 (9)	0		9 (81.8)

^a^Results were considered statistically significant when *p*-values were less than 0.05.

^b^*t*-test.

^c^chi-square test.

^d^Fisher’s exact test.

Abbreviation: HER2, human epidermal growth factor receptor 2; IHC, immunohistochemistry; DISH, dual color *in situ* hybridization.

### Prognosis

The median observation period was 831.5 days (range: 9–5741 days). The overall number of events was 124 cases (50%), and the number of events according to pathologic TNM stage was 6 cases (9.2%) in stage I, 8 cases (19.0%) in stage II, 35 cases (58.3%) in stage III, and 75 cases (92.6%) in stage IV. The number of events is summarized according to HER2 status and pathologic TNM stage in Table 2.

**Table 2:** Number of events among the 248 enrolled patients according to HER2 status and pathologic TNM stage

Pathologic TNM stage, % (number of events/n)	HER2 status			Total (*n* = 248)
	Heterogeneity (*n* = 25)	Homogeneity (*n* = 11)	Negative (*n* = 212)	
I	40.0 (2/5)	0 (0/0)	6.7 (4/60)	9.2 (6/65)
II	25.0 (1/4)	0 (0/2)	19.4 (7/36)	19.0 (8/42)
III	36.4 (4/11)	66.7 (2/3)	63.0 (29/46)	58.3 (35/60)
IV	100 (5/5)	100 (6/6)	91.4 (64/70)	92.6 (75/81)
Total	48.0 (12/25)	72.7 (8/11)	49.1 (104/212)	50.0 (124/248)

Abbreviation: HER2, human epidermal growth factor receptor 2.

### Patients with stage III and IV disease

Given the small number of events reported in patients with stage I and II disease and the administration of targeted HER2 therapy to patients with advanced and recurrence GC, we examined the prognosis of the 141 patients with stage III and IV disease. Trastuzumab-based chemotherapy was administered for two patients with recurrent HER2 positive GC, one of whom had HER2 homogeneity and one HER2 heterogeneity. The number of cycles of trastuzumab-based chemotherapy was 12 cycles for the HER2 homogeneity patient and 3 cycles for the HER2 heterogeneity patient. The clinicopathological characteristics of the 141 patients with stage III and IV disease, according to HER2 status, are summarized in Table 3. Tumors classified as intestinal type, based on Lauren classification, were significantly more frequent (*p* = 0.021) in HER2 positive compared with HER2 negative disease. Pathological subtypes, also based on Lauren classification, were not significantly different between the HER2 heterogeneity group and the HER2 homogeneity group.

**Table 3:** Clinicopathological characteristics of the 141 enrolled patients with stage III and IV disease, according to HER2 status

Variables	HER2 positive		Heterogeneity vs. Homogeneity *p*-value^a^	HER2 status		Positive vs. Negative *p*-value^a^
	Heterogeneity (*n* = 16)	Homogeneity (*n* = 9)		Positive (*n* = 25)	Negative (*n* = 116)	
Age (years)			0.39^b^			0.25^b^
Median	70.5	66.7		69.0	66.5	
Range	47–84	51–81		47–84	38–87	
Advanced age, % (*n*)			0.18^c^			0.34^c^
≥65 years	81.3 (13)	55.6 (5)		72.0 (18)	62.1 (72)	
Sex, % (*n*)			0.14^c^			0.43^c^
Male	62.5 (10)	88.9 (8)		72.0 (18)	63.8 (74)	
Operative method, % (*n*)			0.47^d^			0.26^d^
Distal gastrectomy	68.8 (11)	55.6 (5)		64.0 (16)	55.2 (64)	
Total gastrectomy	31.2 (5)	33.3 (3)		32.0 (8)	42.2 (49)	
Proximal gastrectomy	0	11.1 (1)		4.0 (1)	2.6 (3)	
Pathologic TNM stage, % (*n*)			0.085^d^			0.14^c^
III	68.8 (11)	33.3 (3)		56.0 (14)	39.7 (46)	
IV	31.2 (5)	66.7 (6)		44.0 (11)	60.3 (70)	
Lauren classification, % (*n*)			0.83^c^			0.021^c^
Intestinal type	62.5 (10)	66.7 (6)		64.0 (16)	38.8 (45)	
Diffuse type	37.5 (6)	33.3 (3)		36.0 (9)	61.2 (71)	
Depth of tumor invasion, % (*n*)			0.48^d^			0.11^d^
Muscularis propria	0	11.1 (1)		4.0 (1)	3.4 (4)	
Subserosa	18.7 (3)	33.3 (3)		24.0 (6)	7.8 (9)	
Serosa and peritoneal cavity	75.0 (12)	55.6 (5)		68.0 (17)	81.0 (94)	
Adjacent structures	6.3 (1)	0		4.0 (1)	7.8 (9)	
Lymphatic invasion, % (*n*)			0.29^d^			0.53^d^
ly0	0	11.1 (1)		4.0 (1)	2.6 (3)	
ly1	18.7 (3)	0		12.0 (3)	23.3 (27)	
ly2	37.5 (6)	55.6 (5)		44.0 (11)	35.3 (41)	
ly3	43.8 (7)	33.3 (3)		40.0 (10)	38.8 (45)	
Venous invasion, % (*n*)			0.86^d^			0.087^d^
v0	0	0		0	5.2 (6)	
v1	25.0 (4)	22.2 (2)		24.0 (6)	31.0 (36)	
v2	18.7 (3)	33.3 (3)		24.0 (6)	37.9 (44)	
v3	56.3 (9)	44.5 (4)		52.0 (13)	25.9 (30)	

^a^Results were considered statistically significant when *p*-values were less than 0.05.

^b^*t*-test.

^c^chi-square test.

^d^Fisher’s exact test.

Abbreviation: HER2, human epidermal growth factor receptor 2.

### Overall survival

**Figure 1:**
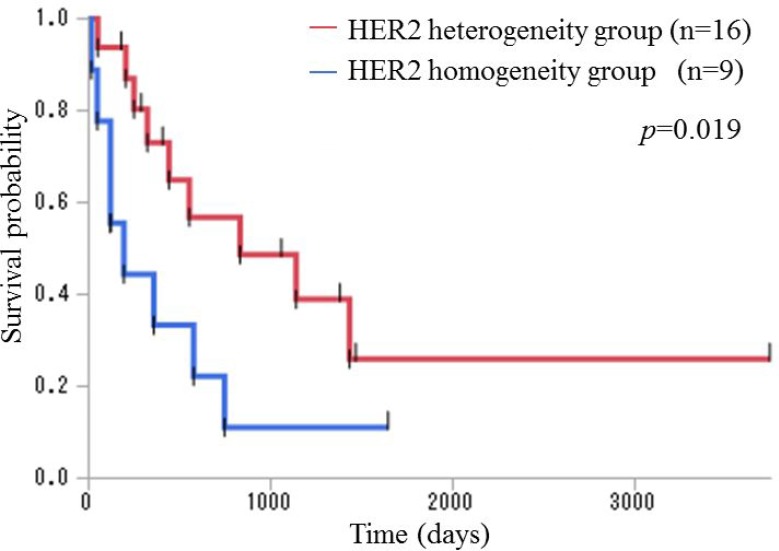
Kaplan–Meier overall survival curves for patients with stage III and IV disease in the HER2 heterogeneity and HER2 homogeneity groups The prognosis of the HER2 homogeneity group was significantly worse than that of the HER2 heterogeneity group (*p* = 0.019; *n* = 9 and *n* = 16, respectively; median OS 193 and 831 days, respectively) using the generalized Wilcoxon test.

**Figure 2:**
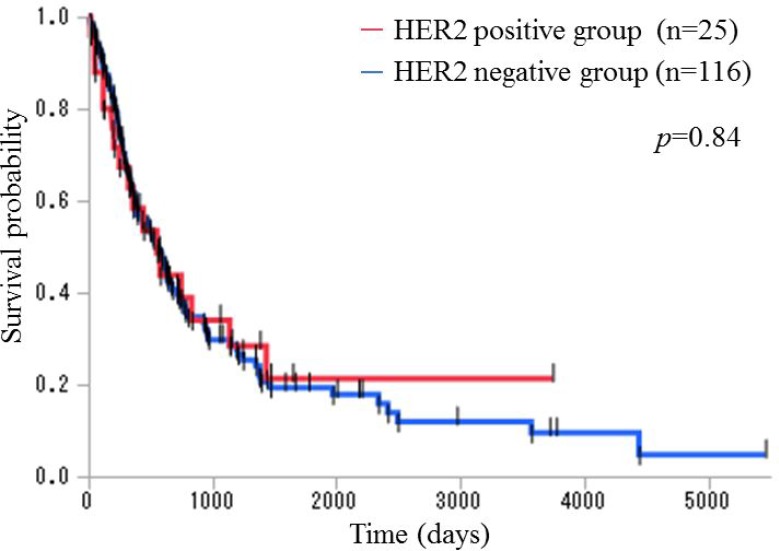
Kaplan–Meier overall survival curves for patients with stage III and IV disease in the HER2 positive and the HER2 negative groups The prognosis was not significantly different between the HER2 positive group and the HER2 negative group (*p* = 0.84; *n* = 25 and *n* = 116, respectively; median OS 552 and 556 days, respectively) using the generalized Wilcoxon test.

We compared overall survival (OS) between the HER2 heterogeneity group and the HER2 homogeneity group. The prognosis of the HER2 homogeneity group was significantly worse than that of the HER2 heterogeneity group (*p* = 0.019; *n* = 9 and *n* = 16, respectively; median OS 193 and 831 days, respectively) using the generalized Wilcoxon test (Figure 1). Subsequently, we compared OS between the HER2 positive group and the HER2 negative group and found no significant difference (*p* = 0.84; *n* = 25 and *n* = 116, respectively; median OS 552 and 556 days, respectively) using the generalized Wilcoxon test (Figure 2).

Excluding the two patients who received trastuzumab-based chemotherapy, the prognosis of the HER2 homogeneity group was significantly worse than that of the HER2 heterogeneity group (*p* = 0.015; *n* = 8 and *n* = 15, respectively; median OS 156 and 1193 days, respectively) using the generalized Wilcoxon test (Supplementary Figure 1). We also compared OS between the HER2 positive group and the HER2 negative group and found no significant difference (*p* = 0.67; *n* = 23 and *n* = 116, respectively; median OS 441 and 556 days, respectively) using the generalized Wilcoxon test (Supplementary Figure 2).

## DISCUSSION

The present study revealed that the prognosis was significantly different between the HER2 heterogeneity group and the HER2 homogeneity group in patients with resectable primary adenocarcinoma of the stomach or gastro-esophageal junction. Overall, the HER2 homogeneity group had a significantly worse prognosis compared with the HER2 heterogeneity group. However, the prognosis was not significantly different between the HER2 positive group and the HER2 negative group.

Our findings show that HER2 homogeneity is associated with a significantly worse prognosis because of the presence of relatively large amounts of HER2 positive components compared with HER2 heterogeneity. Subsequently, the lack of a significant difference in prognosis between the HER2 positive group and the HER2 negative group may be explained by the observation that HER2 positive GC is primarily associated with HER2 heterogeneity. In the present study, the HER2 heterogeneity group accounted for 69.4% of the HER2 positive group, which is within the range of previous reports (45%–79%) [25–28]. The conflicting HER2 prognostic values between GC and BC can be reasonably explained by the markedly different frequency of HER2 heterogeneity between HER2 positive GC and BC. It is unclear whether the role of HER2 as a biomarker of poor prognosis in GC might be to the result of this difference in frequency, whereby the prognostic value might be determined by the extent of HER2 heterogeneity in HER2 positive GC. In addition, the pathological subtype may impact prognosis. In diffuse type GC tumors, most of which are poorly differentiated, patients are more likely to have a poorer prognosis compared with intestinal type GC tumors, most of which are well to moderately differentiated. Intestinal type tumors are more frequent in HER2 positive GC compared with diffuse type tumors [41]. In the present study, tumors classified as intestinal type tumors were significantly more frequent in the HER2 positive group compared with the HER2 negative group and accounted for 64.0% of the HER2 positive group and 38.8% of the HER2 negative group. In addition, the frequency of intestinal type tumors was not significantly different between the HER2 heterogeneity group and the HER2 homogeneity group. According to Qiu *et al*. [41], HER2 positivity was not an independent prognostic factor in GC and the evaluation of HER2 positivity combined with Lauren classification provided a better prognostic value. However, in BC, HER2 positivity is frequent in high nuclear grade BC has been shown to be clinically aggressive [42]. Different features of pathological subtypes between HER2 positive GC and BC may also contribute to the conflicting HER2 prognostic values.

Two studies to date have focused on of the effect of HER2 heterogeneity on GC prognosis. Lee *et al*. [26] examined a single institutional cohort of Korean GC patients and found that the HER2 homogeneity group had a significantly worse prognosis, evaluated by disease-free survival, compared with the HER2 heterogeneity group. Although a different evaluation of survival probability was used, the results of the present study support these findings. Kurokawa *et al*. [25] examined a multi-institutional cohort of Japanese GC patients and found no significant difference in OS between the HER2 heterogeneity group and the HER2 homogeneity group. In addition, they demonstrated that the HER2 positive group had a significantly worse prognosis than the HER2 negative group. Although the reason for the inconsistency between the Kurokawa *et al*. study and our results is unclear, the frequency of events was 78.0% (110/141) among patients with stage III–IV GC disease in the present study. Therefore, we considered the prognosis of most cases in the present study to have been accurately evaluated, and the subsequent results are justifiable.

Improved understanding of the molecular biology of GC and the development of targeted molecular therapy is likely to improve the prognosis of GC patients [43, 44]. Trastuzumab, a monoclonal antibody targeting HER2, is used in the treatment of patients with HER2 positive, inoperable, locally advanced, recurrent, or metastatic GC, although individualized treatment for GC according to HER2 status has not been done. However, HER2 positive GC patients frequently develop resistance to trastuzumab [45, 46] through a mechanism that remains poorly understood, although intratumoral heterogeneity may represent one cause of cancer treatment resistance [47, 48]. According to Lee *et al*. [8], intratumoral HER2 heterogeneity had a poorer treatment response to trastuzumab and was associated with a worse prognosis in patients with HER2 positive metastatic BC. In patients with HER2 positive GC, the evaluation of treatment response to trastuzumab, according to HER2 status, is therefore warranted.

Regarding the processing of pathological specimens, the importance of sustainable quality control is emphasized in the recommendations for HER2 testing in BC by the American Society of Clinical Oncology/College of American Pathologists guidelines [49]. HER2 positivity rates were unaffected by the length of the storage period, indicative of the proper management of pathological samples in our institution.

Our study had some limitations, including the small sample, retrospective design. Second, the data were derived from a single institution, meaning that the interpretation of the results must be generalized with caution. Third, trastuzumab-based chemotherapy was administered to two patients with recurrent HER2 positive GC among the 141 patients with stage III and IV disease. The exclusion of these two patients extended the median OS of the HER2 homogeneity and HER2 positive groups, and the differences in OS increased between the HER2 homogeneity group and the HER2 heterogeneity group as well as between the HER2 positive group and the HER2 negative group. We assume that trastuzumab is more effective in HER2 homogeneity patients and less effective in HER2 heterogeneity patients, indicating that it may not be possible to observe differences in the effect of trastuzumab in HER2 homogeneity BC patients receiving postoperative trastuzumab administration, although this should be evaluated in future studies.

In summary, the present study indicates that prognostic values may differ according to HER2 status, with HER2 homogeneity patients having a worse prognosis compared with HER2 heterogeneity patient. Extrapolations from the present study may be explained by the difference between BC and GC in the clinical implications of HER2 as a biomarker of a poor prognosis. With respect to HER2 heterogeneity and homogeneity in GC, a more precise prognostic prediction may be available for HER2 positive patients. Moreover, responsiveness to anti-HER2 targeted agents in GC may have a potential to vary between HER2 heterogeneity patients and HER2 homogeneity patients. Therefore, well-structured, prospective studies are required to evaluate the prognosis or responsiveness to anti-HER2 targeted agents of HER2 heterogeneity in GC patients, distinct from HER2 homogeneity.

## MATERIALS AND METHODS

### Subjects

This retrospective cohort study included Japanese patients with primary adenocarcinoma of the stomach or gastro-esophageal junction who underwent surgical resection at the Kurume University Medical Center, Japan, between July 2000 and December 2012. The 258 consecutive patients were pathologically confirmed to have adenocarcinoma of the stomach or gastro-esophageal junction. Of the 258 patients, two patients diagnosed with remnant GC and eight patients who had received preoperative chemotherapy were excluded from the study. Postoperative adjuvant chemotherapy is indicated for pathologic TNM stage II and stage III GC. For GC patients with pathologic TNM stage IV or with recurrent disease and whose general condition and major organ functions are preserved, chemotherapy is also indicated. In addition, after March 2011, trastuzumab-based chemotherapy was considered in patients with recurrent HER2 positive GC. None of the 248 enrolled patients had received trastuzumab therapy or radiotherapy prior to surgery. This study was approved by the ethics committee of Kurume University (no. 13128) and conducted in accordance with the principles of the Declaration of Helsinki.

### Clinical variables

The following clinical data were obtained from the patients’ medical records: age at the time of surgery, advanced age (defined as ≥65 years), sex, and operative method (distal gastrectomy, total gastrectomy, proximal gastrectomy, or pylorus-preserving gastrectomy).

### Pathological variables

All cases with resected surgical specimens were retrieved and each slide was reviewed by two independent pathologists (M.M. and R.Y.). Pathological variables were evaluated by consensus of the two pathologists. All surgically resected tissue specimens were fixed in 10% buffered formalin, and formalin fixation time was 6 hours–72 hours. Tissue specimens were embedded in paraffin and processed routinely, and 4-μm sections were stained with hematoxylin and eosin (H&E). Pathologic TNM stage, pathological classification, depth of tumor invasion, lymphatic and venous invasion, and HER2 status were evaluated. The International Union against Cancer/TNM system was applied to classify pathologic TNM stage [50, 51]. Pathological classification was based on Lauren classification (intestinal type or diffuse type) [52]. Depth of tumor invasion (mucosa, submucosa, muscularis propria, subserosa, serosa and peritoneal cavity, or adjacent structures), lymphatic invasion (ly0, ly1, ly2, or ly3), and venous invasion (v0, v1, v2, or v3) were classified in accordance with the Japanese classification of gastric carcinoma [53].

### HER2 status

HER2 status determined in the pathological samples by immunohistochemistry (IHC) and dual color *in situ* hybridization (DISH). An anti-HER2/neu rabbit monoclonal primary antibody (clone 4B5, Ventana, Tucson, AZ, USA) was used for IHC. HER2 and chromosome 17 probes were detected using two-color chromogenic *in situ* hybridization in formalin-fixed, paraffin-embedded tissue specimens in accordance with the manufacturer’s protocol. IHC staining and a HER2 DISH DNA probe cocktail assay were performed using a fully automated Ventana Benchmark XT staining system (Ventana, Tucson, AZ, USA). Antigen retrieval was carried out by heating the sections in EDTA (pH 8.5) in accordance with the manufacturer’s protocol. IHC staining was classified as a score of 3+, 2+, 1+, or 0 to evaluate the degree of HER2 protein overexpression using the HER2 scoring system [51]. HER2 DISH was classified as positive or negative with respect to HER2 gene status by calculating the ratio of the HER2/chromosome 17 centromere (CEN17); a HER2/CEN17 ratio of ≥2 was defined as HER2 DISH positive and a HER2/CEN17 ratio of <2 was defined as HER2 DISH negative.

**Figure 3:**
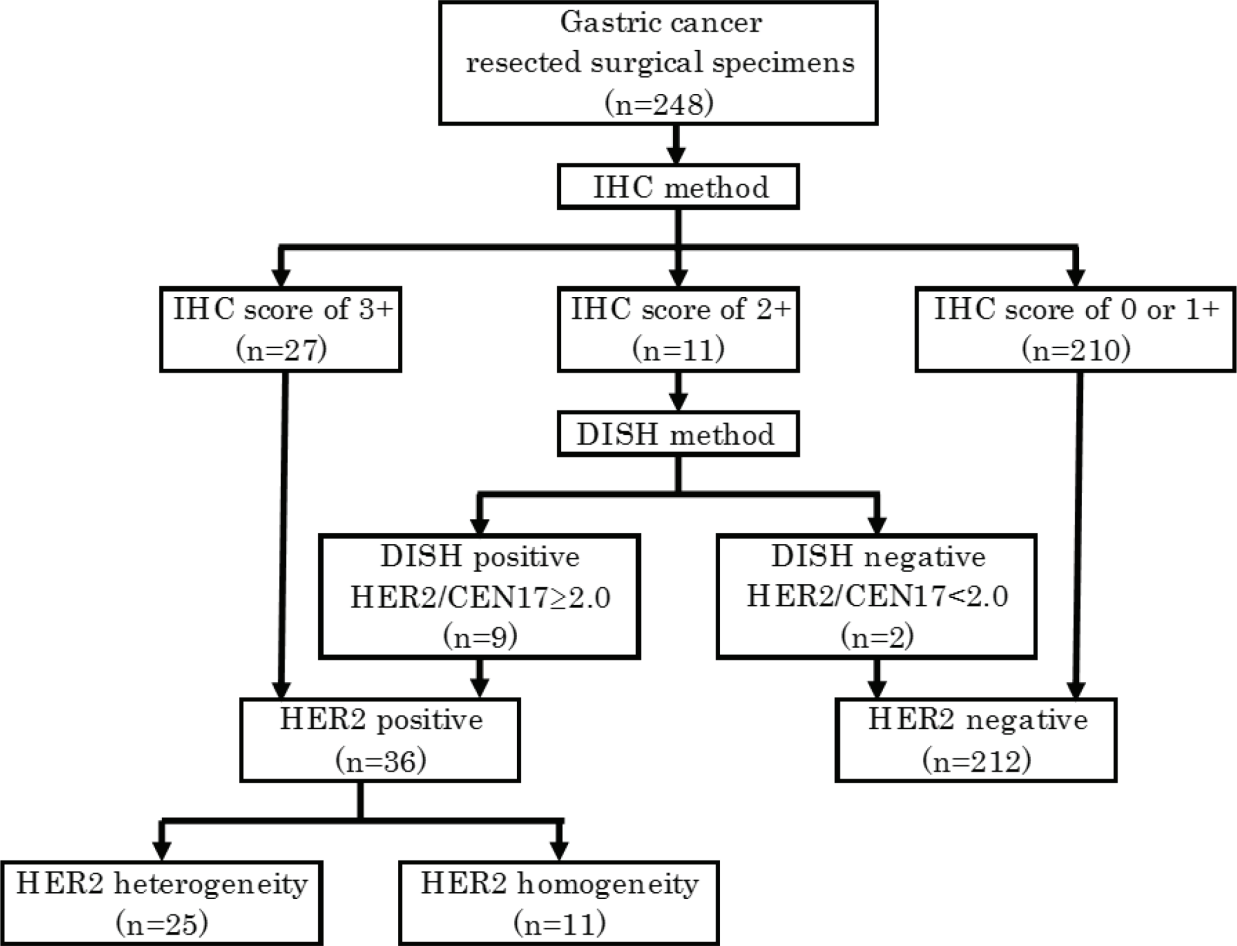
Classification of HER2 status

HER2 status, which was classified as HER2 positive or negative, was assessed with using the IHC score and the HER2/CEN17 ratio. HER2 positive or negative status was then classified in accordance with the Japanese Society of Pathology HER2 pathological diagnosis guidelines GC. Samples with an IHC score of 3+ were defined as HER2 positive, 0 or 1+ were defined as HER2 negative, and 2+ were defined as “HER2 equivocally”. For the latter samples, additional HER2 gene status was evaluated, with HER2 DISH positive samples defined as HER2 positive and HER2 DISH negative samples defined as HER2 negative (Figure 3).

HER2 positive samples were classified into two categories: HER2 heterogeneity or homogeneity. HER2 heterogeneity was defined as 10%–90% of tumor cells showing HER2 protein overexpression in samples with an IHC score of 3+ and or an IHC score of 2+ with DISH positive status. HER2 homogeneity was defined as >90% of tumor cells showing HER2 protein overexpression in samples with an IHC score of 3+. To evaluate quality control of the surgically resected samples in our institution, we divided the cases into two terms by date of the surgical resection with the former term from July 2000 to December 2006 and the latter term from January 2007 to December 2012. We then compared HER2 positivity rates between samples from the two terms using the chi-square test.

### Statistical analysis

The study endpoint was OS, which was defined as in the number of days between the date of GC surgery and the date of death from any cause or last follow-up. The vital status of patients was verified through patients’ medical records in May 2016. OS was estimated using the Kaplan–Meier method, and the differences in OS between the subgroups were compared using the generalized Wilcoxon test. Patients’ characteristics were compared using the chi-square test or Fisher’s exact test for discrete variables, and the *t*-test for continuous variables. Results were considered statistically significant when *p*-values were <0.05. Statistical analyses were conducted using JMP^®^ 13 software (SAS Institute Inc., Cary, NC, USA).

## SUPPLEMENTARY MATERIALS FIGURES AND TABLES


