# Supplementary material for: Prognostic implications of HER2 heterogeneity in gastric cancer
Source: Oncotarget. 2018 Jan 18;9(10):9262–72. doi: 10.18632/oncotarget.24265 (PMC5823644; doi:10.18632/oncotarget.24265)
Supplement: Supplementary file 1 [file oncotarget-09-9262-s001.pdf]

Prognostic implications of HER2 heterogeneity in gastric cancer

SUPPLEMENTARY MATERIALS

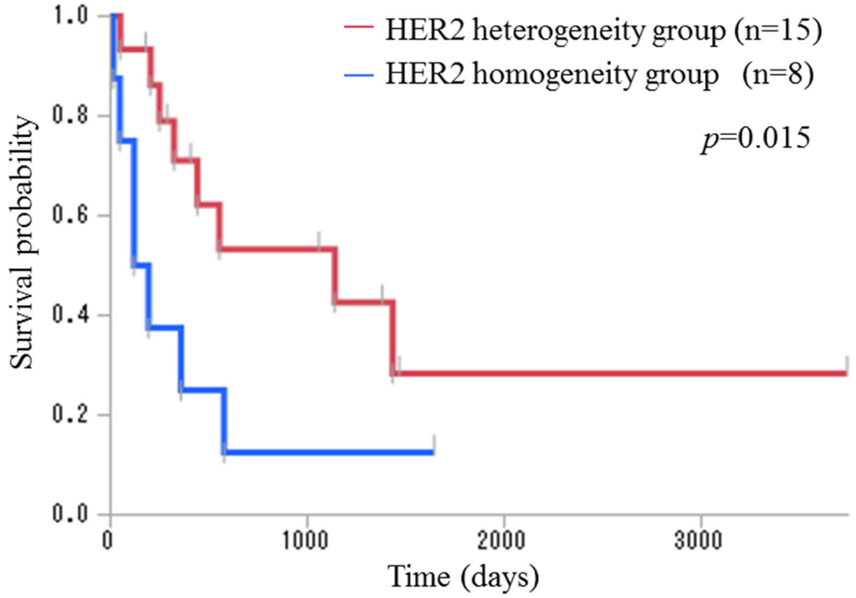

Supplementary Figure 1: Kaplan–Meier overall survival curves for patients with stage III and IV disease, excluding two patients who received trastuzumab-based chemotherapy, in the HER2 heterogeneity and HER2 homogeneity groups. The prognosis of the HER2 homogeneity group was significantly worse than that of the HER2 heterogeneity group ($p = 0.015$; $n = 8$ and $n = 15$, respectively; median OS 156 and 1193 days, respectively) using the generalized Wilcoxon test.

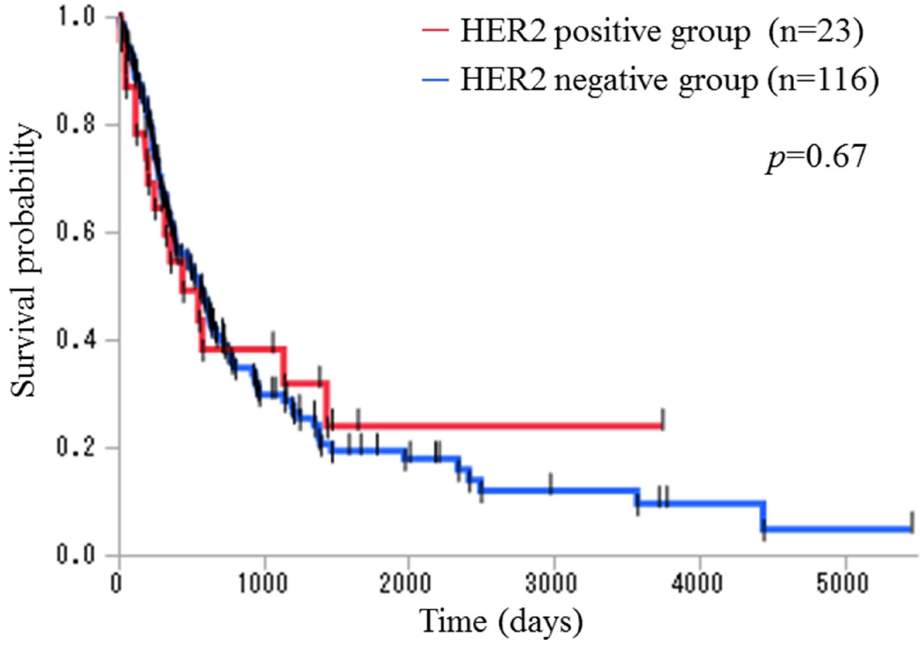

Supplementary Figure 2: Kaplan–Meier overall survival curves for patients with stage III and IV disease, excluding two patients who received trastuzumab-based chemotherapy, in the HER2 positive and the HER2 negative groups. The prognosis was not significantly different between the HER2 positive group and the HER2 negative group ($p = 0.67$; $n = 23$ and $n = 116$, respectively; median OS 441 and 556 days, respectively) using the generalized Wilcoxon test.
